# Machine learning-enhanced assessment of potential probiotics from healthy calves for the treatment of neonatal calf diarrhea

**DOI:** 10.3389/fmicb.2024.1507537

**Published:** 2024-12-09

**Authors:** Yuting Zhai, Miju Kim, Peixin Fan, Sharath Rajeev, Sun Ae Kim, J. Danny Driver, Klibs N. Galvão, Christina Boucher, Kwangcheol C. Jeong

**Affiliations:** ^1^Department of Animal Sciences, University of Florida, Gainesville, FL, United States; ^2^Emerging Pathogens Institute, University of Florida, Gainesville, FL, United States; ^3^Department of Food Science and Biotechnology, Kyung Hee University, Seoul, Republic of Korea; ^4^Department of Food Science and Biotechnology, Ewha Womans University, Seoul, Republic of Korea; ^5^Department of Large Animal Clinical Sciences, University of Florida, Gainesville, FL, United States; ^6^Department of Computer and Information Science and Engineering, University of Florida, Gainesville, FL, United States

**Keywords:** neonatal calf diarrhea, gut microbiome, machine learning, probiotics, host specificity

## Abstract

Neonatal calf diarrhea (NCD) remains a significant contributor to calf mortality within the first 3 weeks of life, prompting widespread antibiotic use with associated concerns about antimicrobial resistance and disruption of the calf gut microbiota. Recent research exploring NCD treatments targeting gut microbiota dysbiosis has highlighted probiotic supplementation as a promising and safe strategy for gut homeostasis. However, varying treatment outcomes across studies suggest the need for efficient treatment options. In this study, we evaluated the potential of probiotics *Limosilactobacillus reuteri*, formally known as *Lactobacillus reuteri*, isolated from healthy neonatal calves to treat NCD. Through *in silico* whole genome analysis and *in vitro* assays, we identified nine *L. reuteri* strains, which were then administered to calves with NCD. Calves treated with *L. reuteri* strains shed healthy feces and demonstrated restored gut microbiota and normal animal behavior. Leveraging a machine learning model, we evaluated microbiota profiles and identified bacterial taxa associated with calf gut health that were elevated by *L. reuteri* administration. These findings represent a crucial advancement towards sustainable antibiotic alternatives for managing NCD, contributing significantly to global efforts in mitigating antimicrobial resistance and promoting overall animal health and welfare.

## 1 Introduction

Neonatal calf diarrhea (NCD) is one of the most common causes of death in preweaning beef calves, posing significant challenges to calf health and farm economics (USDA, 2010). NCD can cause acute gastroenteritis, central nerve system depression, cardiac arrhythmia, and high mortality rates in young calves (Cho and Yoon, 2014). Thus, efficacious treatment and preventive strategies are urgently needed. NCD can be caused by a combination of factors, with infections caused by pathogenic bacteria, viruses, and protozoa being prime contributors (Maier et al., 2022; Meganck et al., 2014; Wei et al., 2020). Environmental stressors and nutritional factors also play pivotal roles in the manifestation and severity of this condition (Bendali et al., 1999; Cho and Yoon, 2014). In the first week after birth, enterotoxigenic *E. coli* (ETEC) infection is the typical cause of NCD, and cryptosporidium and virus infections are the main reasons for NCD from day 5–30, while *Salmonella* infection is the main reason from day 14–60 (CDFA, 2022). In this complex scenario, probiotics have emerged as a promising adjunct to conventional therapeutic approaches, particularly given escalating concerns over antimicrobial-disturbed gastrointestinal microbiota, the rise of antibiotic resistance, and increasing consumer preferences for antibiotic-free animal products (Du et al., 2023; Ji et al., 2018).

Probiotics are administered with the intent of establishing a healthy gut microbiome, which is crucial for enhancing immunity, improving gut integrity, and potentially mitigating the impact of enteric pathogens (Carr et al., 2002; Hill et al., 2014; Suez et al., 2020; Xiao et al., 2024). Despite the growing utilization of probiotics, the efficacy of probiotics in neonatal calves has been variable, reflecting the influence of the specific probiotic strains utilized and the pre-existing health conditions of the animals (Beck et al., 2022; Vitetta et al., 2017). Probiotics exert their beneficial effects through various mechanisms that include competition with pathogens for colonization sites and nutrients, modulation of the host immune response, and the production of antimicrobial peptides (Liu et al., 2021; Mohseni et al., 2021; Plaza-Diaz et al., 2019; Raheem et al., 2021). These effects contribute to the maintenance of gut homeostasis and balance. Despite the widespread public interest in probiotics, the evidence supporting their effectiveness often presents inconsistencies and conflicts. Veiga *et al* highlighted the need to transition from generic probiotics to precision probiotics (Veiga et al., 2020). They outlined the necessary future steps for the development of targeted probiotics, emphasizing strategies that prioritize phenotypic and target-based discovery, along with trials customized to individual needs and responses. This evolving perspective seeks to refine the understanding and application of probiotics, moving beyond a one-size-fits-all approach towards a more customized paradigm.

In the previous study, we investigated the gut microbiota and its association with calf diarrhea (Fan P. et al., 2021). Neonatal calves with abnormal feces, characterized by either watery or hemorrhagic feces, exhibited significantly less bacterial richness compared to those with normal feces. The lower gut bacterial diversity was more pronounced with the escalating severity of fecal abnormalities. Notably, we identified bacterial taxa such as *Christensenellaceae*, *Oscillospiraceae*, *Barnesiella*, *Parabacteroides*, and *Lactobacillus*, which were more abundant in normal feces. Furthermore, *Lactobacillus* strains isolated from healthy calves showed antimicrobial activity against pathogens associated with cattle diarrhea, including *E. coli* K88 and *Salmonella Typhimurium*. These data suggest that a high abundance of *Lactobacillus* in calves may play a pivotal role in reducing NCD. Furthermore, we reported that microbiota composition is shaped by host genetic background and modulated by host–microbe interactions (Fan P. et al., 2020; Fan P. X. et al., 2021). Therefore, we hypothesized that the probiotics derived from the gut microbiota of healthy calves might effectively cure NCD in calves, especially those raised in the same herd because they have relatively small variations in their host genetic background and growth environment.

In this study, we developed probiotics to treat NCD using *Limosilactobacillus* strains isolated from healthy calves. Furthermore, we successfully developed a machine learning (ML) model to identify health-status-associated bacterial taxa and predict calf health indexes. The results indicate a notable conversion of the gut microbiota toward increased gut health indexes.

## 2 Materials and methods

### 2.1 Animal selection and management

The animal study was reviewed and approved by the University of Florida Institutional Animal Care and Use Committee. The calves chosen for probiotic treatment were selected based on the severity of their diarrhea and the ineffectiveness of prior antibiotic treatment.

### 2.2 Whole-genome sequencing and bioinformatics

Whole-genome sequencing (WGS) was carried out on 22 *Lactobacillus reuteri* strains (Supplementary Table S1). The DNA extraction was performed using the DNeasy blood and tissue kit (Qiagen, United States), following the protocol for Gram-positive bacteria. For WGS sequencing, the library was prepared using the Nextera XT sample preparation kit (Illumina, United States), as per the manufacturer’s instructions. The sequencing was conducted using the Illumina MiSeq platform. The sequencing data were processed by trimming with Sickle and assembled with SPAdes (Joshi and Fass, 2011; Prjibelski et al., 2020). Core-genome alignment was performed using Roary (Page et al., 2015). while maximum likelihood phylogenetic trees were generated by IQ-Tree (Nguyen et al., 2015), with ModelFinder used for determining the best tree model and ultrafast bootstrap analysis employing 1,000 replicates.(Kalyaanamoorthy et al., 2017) The resulting tree was visualized using iTol (Letunic and Bork, 2021). Functional inference of the *L. reuteri* strains was conducted via genome-wide functional annotation using eggnog-Mapper (Huerta-Cepas et al., 2017). The identification of antimicrobial resistance genes and virulence factors followed previously established methods (Zhai et al., 2021). A circular genome map was generated for the comparison of the nine selected *L. reuteri* strains, which was analyzed using Proksee (Grant et al., 2023). The bacteriocin gene was identified using BAGEL (van Heel et al., 2018).

### 2.3 *In vitro* characterization for probiotic profiling

Twenty-two strains of *L. reuteri*, along with a reference strain, ATCC53608, isolated from the small intestine of a pig, were evaluated for their probiotic potential through various tests. The viability of the strains was tested in simulated gastrointestinal juice (SGJ) and simulated colonic environment (SCEM). The SGJ was formulated with 0.5% w/v NaCl, 0.5% w/v pepsin, and sterilized water, with the pH adjusted to 2. SCEM was prepared following the method described by Polzin et al. (2013) with a pH of 7. The *L. reuteri* strains were initially grown in MRS (De Man, Rogosa and Sharpe) broth (BD Difco™, United States) under anaerobic conditions overnight, then diluted 100-fold in fresh MRS broth and further incubated anaerobically for 18 h at 37°C. The bacteria were then harvested, washed with PBS, and incubated in SGI for 2 h and in SCEM for 12 and 24 h. Bile salt tolerance was assessed by culturing each strain in MRS broth containing 0.1, 0.2, and 0.3% bile salt (Sigma-Aldrich, United States), followed by incubation at 37°C under anaerobic conditions for 4 h. Acid tolerance was tested in MRS broth at pH = 2, culturing *L. reuteri* strains for 2 h. Lastly, a bacterial competition assay was conducted against *E. coli* K88 with slight modification (Massip et al., 2019). *L. reuteri* strains were co-cultured with *E. coli* K88 at a 1:1 ratio in MRS broth for 24 h. To evaluate the suppression activity of *L. reuteri*, bacterial cell counts were performed before and after incubation using serial dilution and the plating method. The total number of *E. coli* K88 was counted on LB agar after incubation at 37°C for 24 h.

### 2.4 Oral administration and fecal sample collection

Three diarrheic calves were administered orally with a mixture of nine *L. reuteri* strains, receiving approximately 10^9^ colony-forming units (CFUs) daily per calf. To prepare this probiotic formulation, the overnight cultures of each *L. reuteri* strain were diluted 100-fold in fresh MRS broth and allowed to grow for 16 h. Subsequently, the bacteria were harvested, washed with PBS, and resuspended in 1 mL of 30% glycerol. The probiotic products were stored at −80°C until administrated. For administration, probiotics were mixed with 9 mL of water in a syringe to orally feed. The morphology of calf feces was documented through photographs. For tracking changes in the gut microbiome, fecal samples were collected daily from the rectal anal junction using sterile swabs during the treatment period and on days 8 and 11 post-treatment using sterile swabs, as described previously (Fan P. et al., 2021).

### 2.5 16S rRNA sequencing and microbiome analysis

16S rRNA sequencing was conducted to analyze the microbiome composition. Thawed fecal samples were homogenized on ice, and 500 μL of each sample underwent DNA extraction using the QIAamp PowerFecal DNA kit as per the manufacturer’s protocol (Qiagen, United States). Subsequently, the V4 region of the 16S rRNA gene was amplified and sequencing was carried out on the MiSeq platform (2 × 250 bp). The resulting sequencing data underwent analysis using version 2 of the Quantitative Insights into Microbial Ecology (QIIME 2) pipeline (Bolyen et al., 2019). Paired-end reads were imported, and initial base quality was evaluated through the Interactive Quality Plot. Quality control of sequences was performed using the Divisive Amplicon Denoising Algorithm (DADA2) pipeline integrated into QIIME 2, encompassing steps such as filtering low-quality reads, denoising, merging pair-ended reads, and discarding chimeric reads. A phylogenetic tree was constructed utilizing the align-to-tree-mafft-fasttree pipeline from the q2-phylogeny plugin of QIIME 2. Sequencing depth was standardized to 1,1,043 sequences per sample. Evaluation of microbial diversity was conducted using the Shannon index and Bray–Curtis distance via the core-metrics-phylogenetic method. All amplicon sequence variants (ASVs) were taxonomically classified into bacterial taxa using the q2-feature-classifier plugin of QIIME 2 in conjunction with the SILVA 132 database.^1^ The relative abundance of bacterial taxa was determined by dividing the bacterial abundance by the sequencing depths.

### 2.6 Co-occurrence network analysis

To anticipate bacteria–bacteria interactions within the gut microbial community at various stages, co-occurrence patterns of bacterial genera were examined within each stage (early stage: day 0–1, middle stage: day 4–5, late stage: day 8–11) using pairwise Spearman’s rank correlations (rs) based on relative bacterial abundance. The Spearman’s rank correlations were assessed using the Hmisc package of R 4.3.1 (R: A Language and Environment for Statistical Computing, 2022). A significant rank correlation between two genera (*p* < 0.05) indicated a co-occurrence event. The network was depicted using the Fruchterman Reingold layout within the interactive platform Gephi 0.10.1 (Bastian et al., 2009; Fruchterman and Reingold, 1991). In the network, nodes represent different bacterial genera, while edges signify significant correlations between nodes. Node size reflects the degree of connection, and edge thickness indicates the strength of correlation.

### 2.7 Machine learning model construction and health-index prediction

To explore the relationship between calf health status and gut microbiome composition, including specific bacterial taxa, a machine learning approach was introduced. The random forest algorithm was employed for classifying microbiome differences (Ho, 1995). Initially, the model was trained using microbiome data of 91 calves (74 healthy and 17 with diarrhea) collected previously (Fan P. et al., 2021). The relative abundance of bacterial taxa and the health status of the calf were used as training data. Three-fold cross-validation was applied to optimize model performance. We employed a feature importance function to pinpoint bacterial taxa that were crucial for health status classification. Subsequently, taxa with an importance value greater than 0 (including 150 taxa) were selected to refine and improve the model’s accuracy. After developing the model, it was then applied to predict the health status of calves in the current study. The model calculated a probability score for each calf based on the relative abundance of its gut bacterial taxa. A probability score close to 1 indicated a likelihood of healthy gut microbiota in the calf. The procedure was carried out using the Scikit-learn package and visualized using the Seabron package^2^ in Python (Pedregosa et al., 2011).

### 2.8 Statistical analysis

Specific statistical analyses were performed on each *in vitro* experiment to evaluate their outcomes comprehensively. The viability of strains in SGI was assessed using one-way ANOVA followed by Tukey’s multiple comparisons test, while the viability in an acidic condition (pH = 2, MRS media) was examined through one-way ANOVA followed by Dunnett’s multiple comparisons test. The viability under varying bile salt concentrations was analyzed via two-way ANOVA followed by Tukey’s multiple comparisons test, while the duration of strains in SCEM was tested using two-way ANOVA followed by Dunnett’s multiple comparisons test. Growth inhibition ability against ETEC K88 was determined by one-way ANOVA followed by Dunnett’s multiple comparisons test in a competitive assay, with *L. reuteri* ATCC53608 as the control for comparison. These statistical analyses were conducted using GraphPad Prism version 10.1.1 for MacOS, provided by GraphPad Software based in Boston, Massachusetts, United States.^3^

## 3 Results

### 3.1 Genomic similarity of potential probiotics isolated from healthy calves

**Figure 1:**
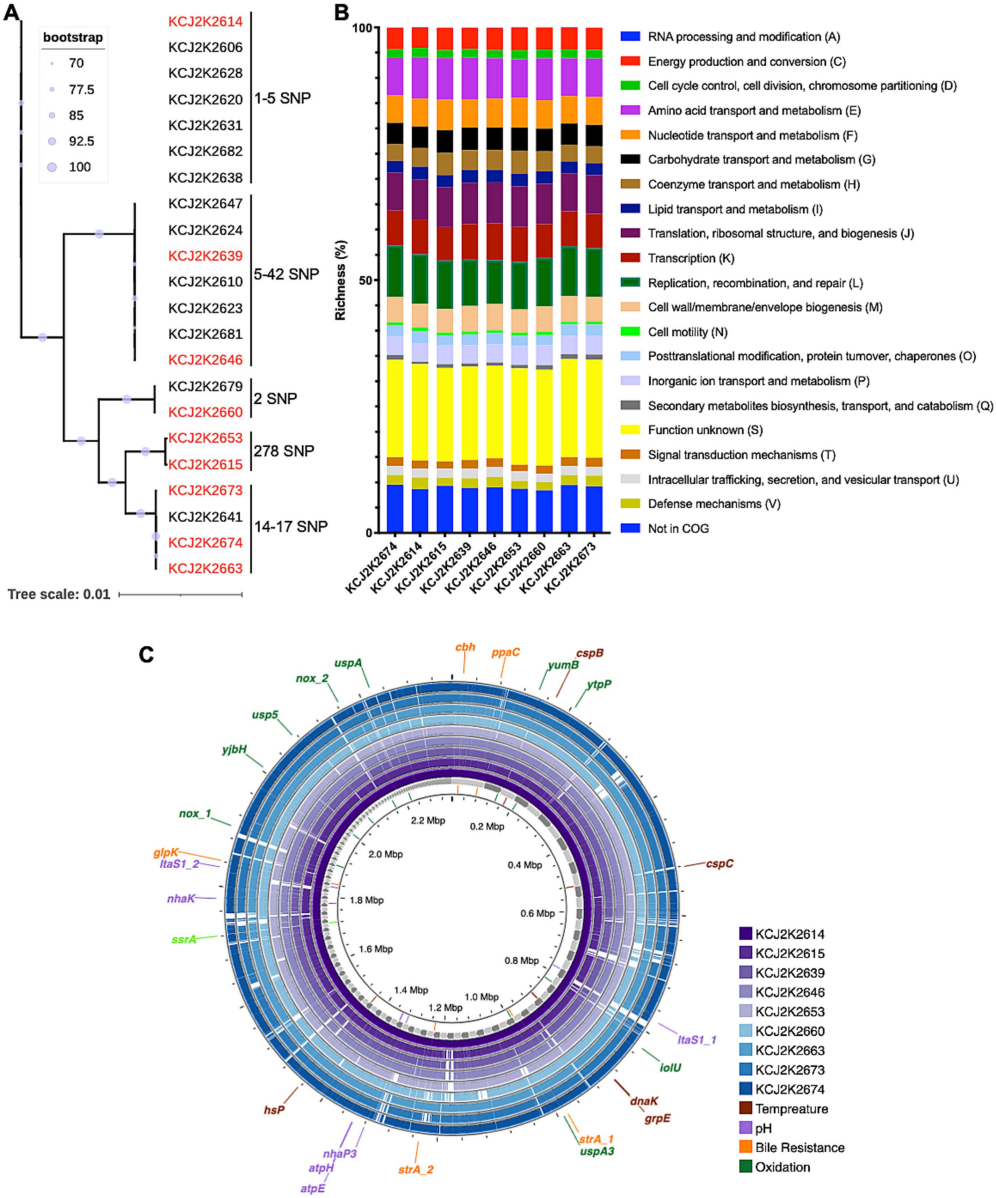
Genetic homogeneity and host-specific phylogeny of *L. reuteri* strains isolated from healthy neonatal calves. **(A)** Phylogenetic relatedness of *L. reuteri* strains form healthy neonatal calves. Bootstrap values indicate the confidence of each branch, with single nucleotide polymorphisms (SNPs) annotated. Nine strains (highlighted in red) were selected for further investigation. **(B)** Clusters of Orthologous Groups (COG) categories across nine selected strains. The y-axis depicts the proportion of genes within various COG functional groups, reflecting the relative richness of each cluster within a specific strain. **(C)** Comparative genome visualization of nine selected strains, with KCJ2K2614 as the reference. Each individual genome is denoted by a different color. Genes that confer resilience to environmental stressors are annotated on the outermost circle.

*Limosilactobacillus reuteri* was identified as the predominant species in the normal feces of healthy claves in the previous study, exhibiting significant antimicrobial activity against diarrheagenic *E. coli* and *S. typhimurium* (Marianelli et al., 2010; Schwaiger et al., 2023). In this study, we aimed to assess the therapeutic potential of *L. reuteri* strains for the treatment of neonatal calf diarrhea. To understand the genomic characteristics of these strains, we conducted WGS and performed a phylogenetic analysis on 22 *L. reuteri* strains isolated from healthy calves (Supplementary Table S1). As shown in Figure 1A, a remarkable degree of genetic similarity was observed among the strains. Despite this overall genomic similarity, distinct genomic clusters emerged, revealing nine discernible clusters with 1-278 single nucleotide polymorphisms (SNPs) in each cluster. In order to elucidate the metabolic pathways, we selected representative 9 strains highlighted in red from each cluster and predicted functional gene categories according to the Cluster of Orthologous Groups (COG) framework. Gene functions involved in genome replication, recombination, repair, RNA processing, and modification exhibited high richness among strains (Figure 1B). The majority of functional gene categories were similar across the strains, with minor variations, possibly reflecting subtle genetic distinctions. Taken together, the similarity in both phylogenetic relatedness and COG profiles among the nine *L. reuteri* strains suggest that clonal variants of *L. reuteri* strains prevail in animals on the same farm, with likely similar functionalities within the gastrointestinal tract of calves.

### 3.2 Functional annotation of *Lactobacillus reuteri* genomes

To identify differences in genome structure, we compared the selected genomes. Consistent with the phylogenetic tree analysis (Figure 1A), these genomes exhibited a high degree of genomic similarity with minor gene addition and deletion (Figure 1C). Genes associated with probiotic functions are shown in Figure 1C and listed in Supplementary Table S2. The *enlA* gene encoding bacteriocin enterolysin A was encoded in all genomes. Genes associated with bile salt resistance, including *cbh*, *ppaC*, *glpK*, and *srtA*, were identified, and genes conferring pH tolerance, such as Alkaline phosphatase (*itaS*), F0F1 ATP synthase (*atpE* and *atpH*), and sodium proton antiporters (*nhaK* and *nhaP3*), were also present in all nine genomes. Cold and heat shock proteins, *cspB*, *cspC*, *dnaK*, *grpE*, and *hsp.,* related to temperature stress, were detected in all nine strains. In addition, we identified oxidative stress-related genes in the *L. reuteri* strains, such as *nox*, *yumB*, *iolU*, *usp5*, *uspA*, *uspA3*, *ytpP*, and *yjbH*, which contribute to probiotic potential of the *L. reuteri* strains. Furthermore, we annotated the antibiotic resistance genes and the virulence factors in the nine *L. reuteri* genomes, using Comprehensive Antibiotic Resistance Database and the Virulence Factor Database (Alcock et al., 2023; Chen et al., 2005). All nine genomes carried a partial *vanT* gene in vanG cluster, 34.49% identify in 52.67% of total gene length, which exerts glycopeptide resistance (Supplementary Table S2). However, virulence genes were not found in the evaluated genomes.

### 3.3 Host-origin-associated genomic heterogeneity of strains

**Figure 2:**
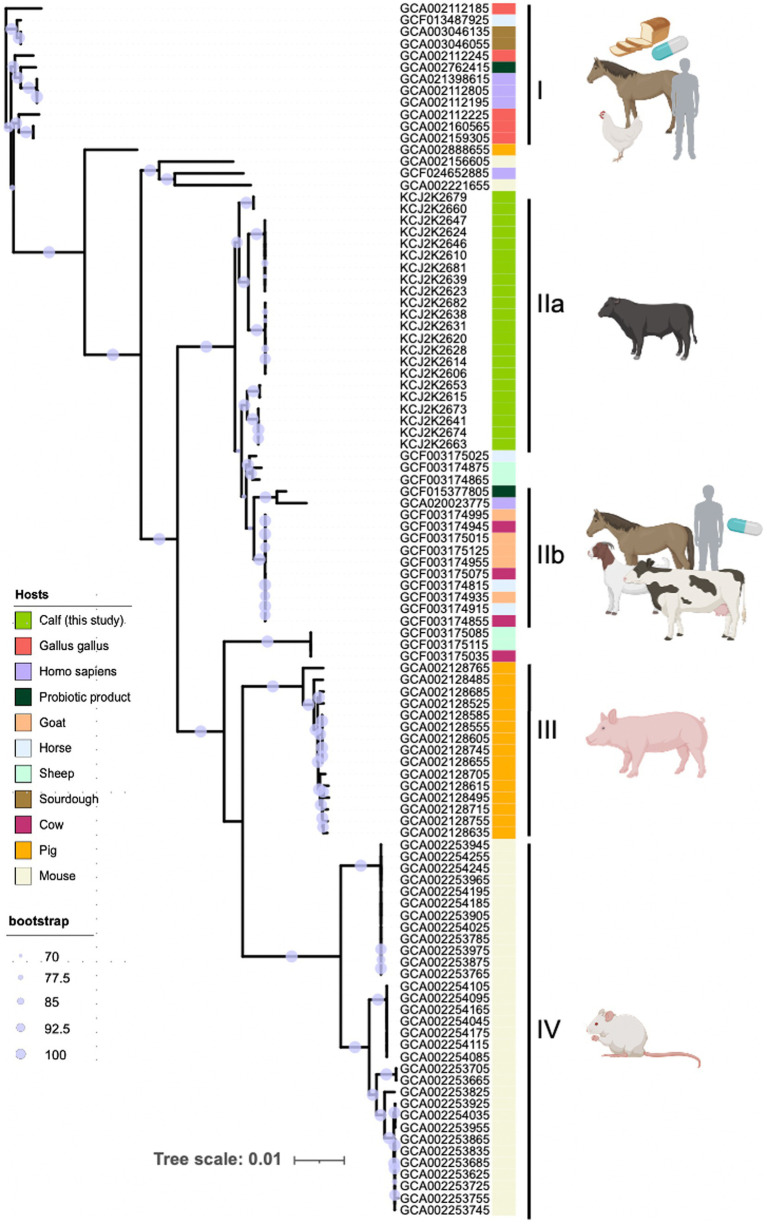
Host-specific phylogeny of *L. reuteri*. The phylogenetic tree included 105 *L. reuteri* genomes—83 from NCBI and 22 from this study—showcasing their genetic relatedness and host-specific grouping. Strains fall into five clades (I, IIa, IIb, III, and IV), reflecting their adaptation to different host niches. Host origins are denoted by colored strips. Node size indicates bootstrap support, with larger nodes signifying greater confidence. The scale represents genetic distance to illustrate evolutionary relationships.

To explore the heterogeneity of *L. reuteri*, we investigated the phylogenetic relatedness of WGS of various *L. reuteri* strains obtained from a range of host sources. We collected 83 *L. reuteri* WGS data originating from horses, sheep, chicken, cow, pig, sourdough, probiotic products, and humans from the NCBI database (Supplementary Table S3) and conducted a core-genome-based phylogenetic analysis (Figure 2). The node confidence is statistically supported by bootstrap values, where a higher value corresponds to a higher statistical support for the node. These 105 genomes formed five distinct clades (I, IIa, IIb, III, and IV). Interestingly, two clades (I and IIb) were clustered with genomes from multiple hosts including human probiotic products, suggesting one type of probiotics was commonly used. Alternatively, a type strain of *L. ruteri* can colonize different hosts. Interestingly, three clades (IIb, III, and IV) were specifically associated with beef calves, pigs, and mice, respectively, indicating these strains have been adapted to specific host niches. These data suggest that *L. ruteri* isolated from beef calves may have enhanced potency to treat NCD in claves.

### 3.4 Probiotic characteristics of *Lactobacillus reuteri* strains

**Figure 3:**
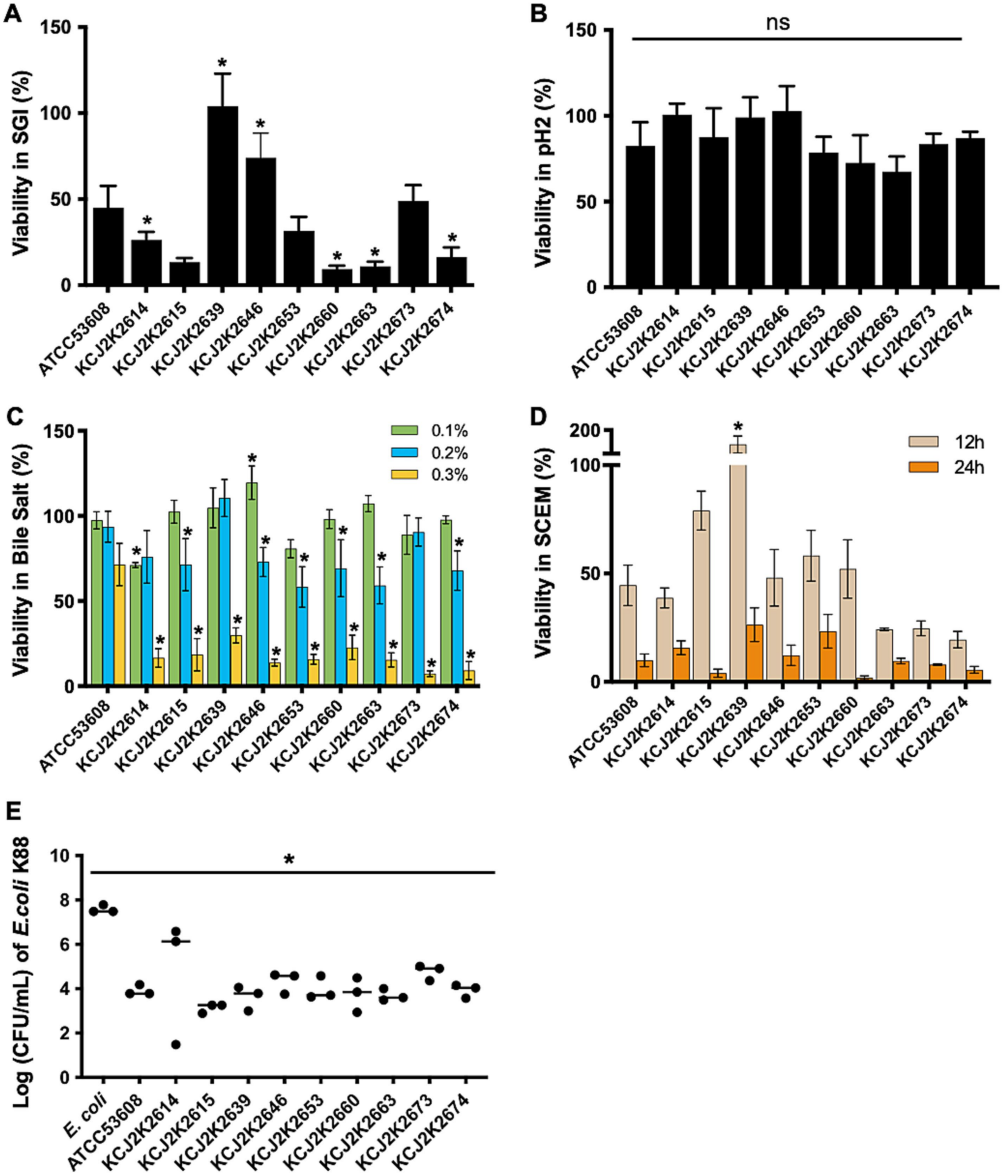
Probiotic potential of *L. reuteri* strains in various conditions. **(A)** The survival of *L. reuteri* strains was tested in simulated gastric conditions at pH 2 for 2 h, comparing their viability to the reference strain *L. reuteri* ATCC53608. **(B)** The viability of *L. reuteri* strains in MRS media at pH 2 for 2 h was measured. **(A,B)** One-way ANOVA was used for statistical comparison against the reference strain. **(C)** The viability of *L. reuteri* strains in bile salts at 0.1, 0.2, and 0.3% for 2 h. Statistical difference was determined against the reference strain using two-way ANOVA. **(D)** The viability of *L. reuteri* strains in a simulated colonic environment at neutral pH over 12 and 24 h. Statistical difference was determined against the reference strain using two-way ANOVA. **(E)** Competition assay to measure the inhibitory effect of *L. reuteri* strains on the growth of ETEC K88 after 24 h of co-culture. Statistical difference was determined against the reference strain using one-way ANOVA. **(A–E)** Significant differences are marked by asterisks for *p*-values less than 0.05.

To assess probiotic potential of the isolates, we conducted *in vitro* analyses, mimicking the conditions of the gastrointestinal (GI) tract. Acid tolerance was evaluated in a simulated gastrointestinal fluid (SGI, pH 2) for 2 h (Figure 3A). Probiotic *L. reuteri* ATCC53608 was used as a reference strain (MacKenzie et al., 2010 Heavens et al., 2011). The survival rate varied among strains, ranging 20 to 100% in SGI. KCJ2K2639, KCJ2K2646, and KCJ2K2673 showed significantly greater survival rates compared to ATCC53608. However, all tested strains showed enhanced survival rate in MRS (pH = 2) compared to SGI, suggesting that complex media composition might provide protection activity at low pH (Figure 3B). Subsequently, we assessed the survival rate of *L. reuteri* strains at different concentrations of bile salts (Figure 3C). All strains showed greater than 50% viability in both 0.1 and 0.2% bile salt, whereas viability decreased at 0.3% bile salt. However, all strains maintained viability, demonstrating their tolerance to high bile salt. Specifically, KCJ2K2646 showed significantly higher viability at 0.1% bile salt. At 0.2%, KCJ2K2614, KCJ2K2639, and KCJ2K2673 showed comparable viability to the reference strain. After 12 h and 24 h of treatment in a simulated colonic environment (SCEM) at pH 7, strains showed varying survival rates (Figure 3D). Particularly, KCJ2K2639 showed outstanding viability with bacterial growth and demonstrated a significantly higher viability compared to the reference strain. To investigate whether the strains may suppress pathogenic *E. coli* growth, a competitive growth assay (Figure 3E) was performed. Co-culturing enterotoxigenic *E. coli* (ETEC) K88, a known diarrheagenic *E. coli* in calves, with *L. reuteri* strains for 24 h resulted in a significant inhibition of *E. coli* K88 growth. Given that the *in vitro* test results demonstrate all 9 tested strains can survive in the GI tract environments and suppress ETEC infection, *L. reuteri* strains have the potential to serve as treatment for NCD in calves.

### 3.5 Probiotic administration cured diarrhea and restored gut microbiota diarrheic calves

**Figure 4:**
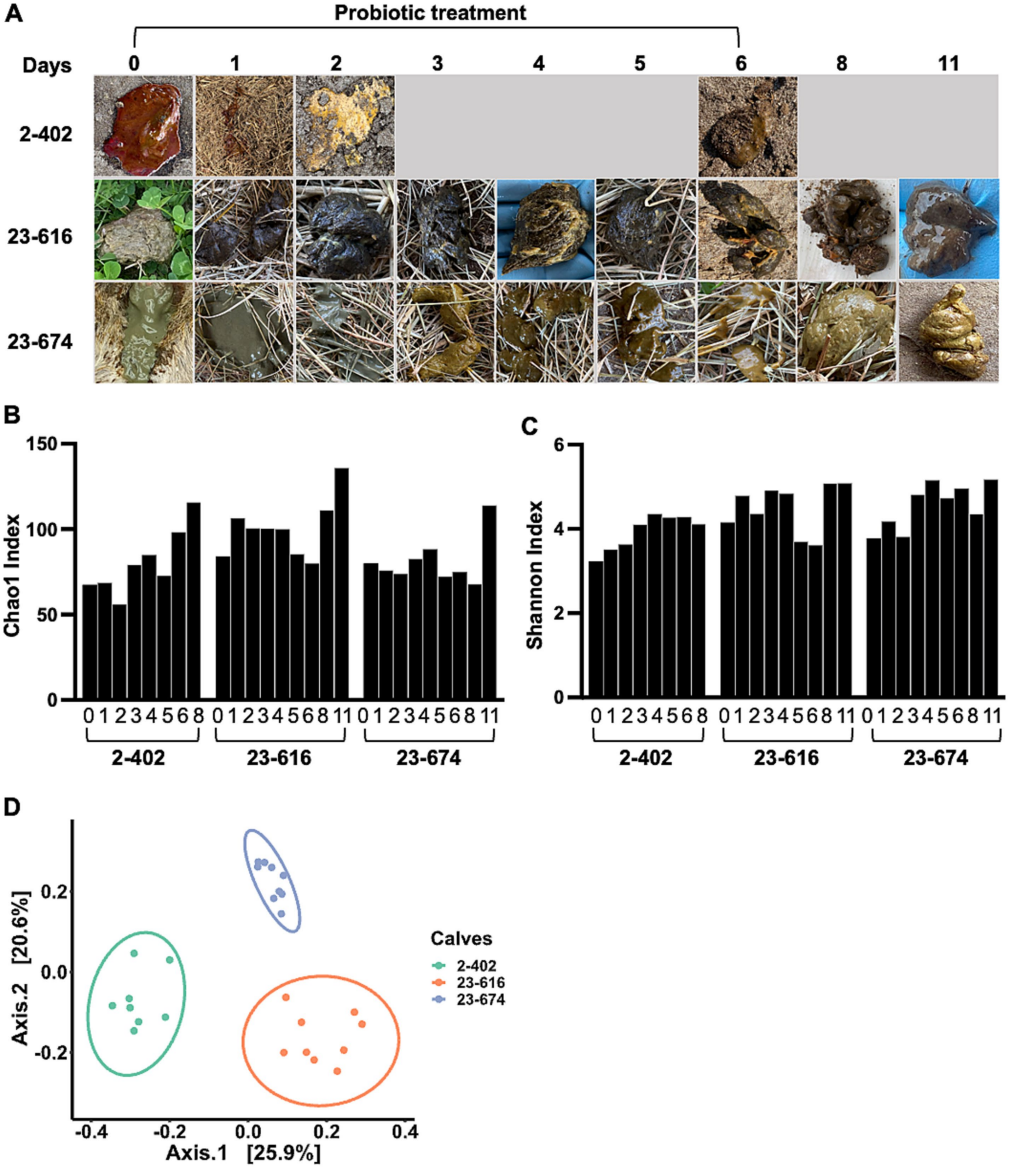
Impact of probiotic treatment on gut microbiota diversity and individual responses in calves. **(A)** The timeline of probiotic treatment and the corresponding sample collection points. Accompanying photographs illustrate the changes in fecal morphology observed in diarrheic calves throughout the course of probiotic treatment (Day 1 – 6). Fecal morphology was recorded on Day 0 prior to probiotic treatment. **(B,C)** An increase in gut microbiota richness and evenness during the treatment period. The Chao1 index **(B)**, highlighting species richness, and the Shannon index **(C)**, reflecting both species abundance and evenness, indicate the development of more diverse and abundant microbial communities in three calves undergoing probiotic treatment. **(D)** The beta-diversity among probiotic-treated calves, analyzed using the Unweighted-Unifrac dissimilarity matrix. The data reveal distinct, individualized microbiome structures among the three calves, underscoring the personalized microbial response to probiotic intervention.

To evaluate probiotic candidates for NCD treatment, a mixture of 9 *L. reuteri* strains was administered to diarrheic calves. Three calves with severe diarrhea were selected by a veterinarian after the failure of antibiotic treatment. Due to the severity of diarrhea, no treatment control was not included to prioritize animal welfare. Probiotics were administrated on Day 0, and feces were collected from the rectal anal junction at specified intervals post-administration. Feces morphology was monitored throughout the treatment period. As shown in Figure 4A, there were noticeable changes in fecal morphology, transitioning from bloody or watery feces to normal brown feces, along with observable improvements in animal behavior, indicating that probiotics were effective for treating diarrhea. Microbiota changes were assessed through 16S rRNA sequencing of feces during treatment. Alpha diversity analysis, comparing the bacterial community richness (Figure 4B) and diversity (Figure 4C) during treatment, revealed increased parameters on Day 8 or 11 across all three calves, as compared to Day 0 (Figures 4B,C). However, distinct microbiota structures were observed among the treated calves (Figure 4D) which was consistent with feces morphology. Given the association between higher bacterial richness and improved gut health (Fan P. et al., 2021), the observed enhancement in alpha diversity indicates the effectiveness of probiotics in restoring gut microbiota in diarrheic calves.

**Figure 5:**
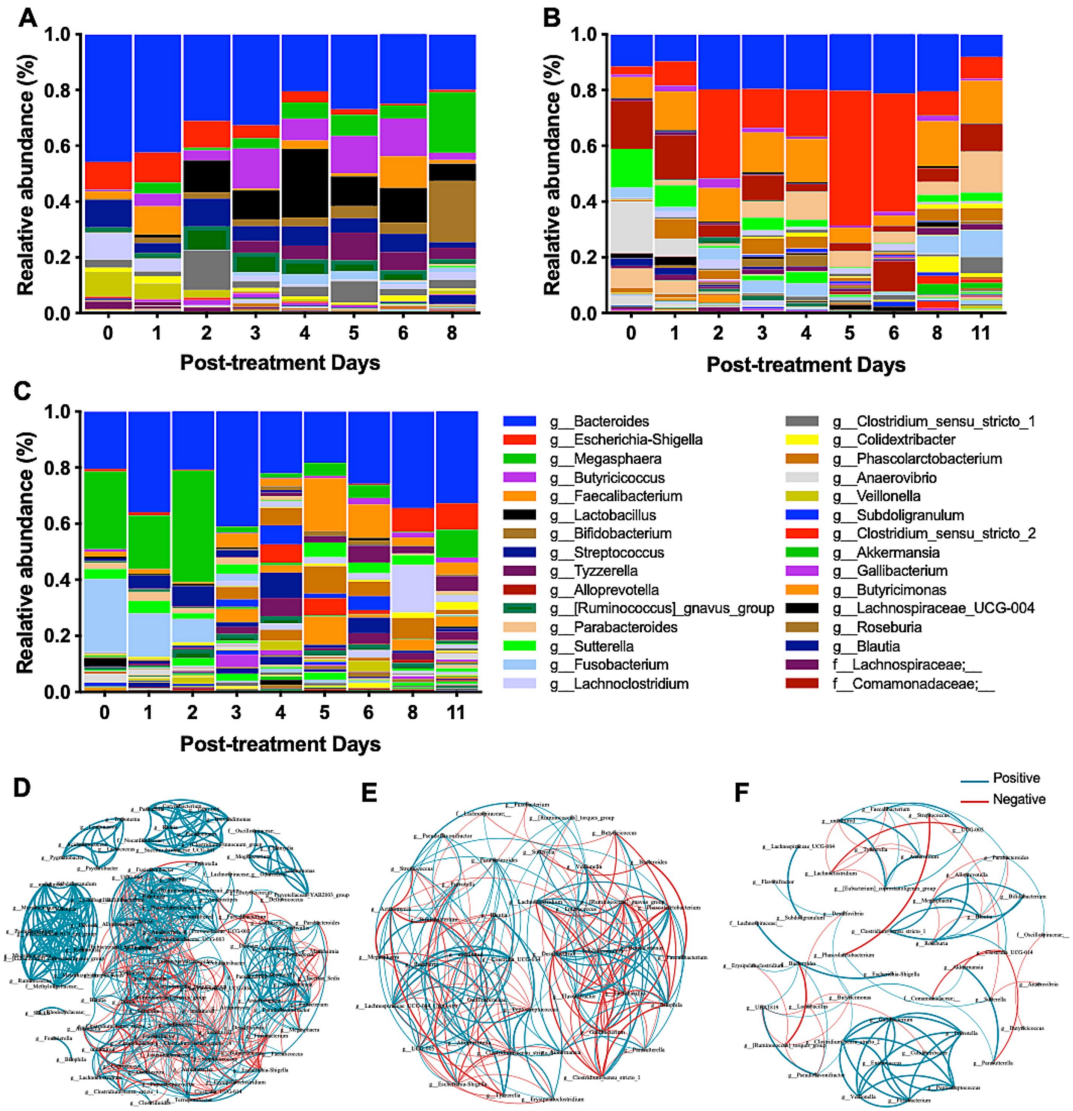
Comprehensive analysis of microbiome diversity and bacterial interactions during probiotic treatment. **(A–C)** The relative abundance of bacterial genera at various time points throughout the probiotic intervention provides insights into the dynamic shifts in microbial populations in response to the treatment. The relative abundance of bacterial genera is presented for individual calve: **(A)** 2-402, **(B)** 23-616, and **(C)** 23-674. **(D–F)** The co-occurrence networks of the microbiome were captured at distinct phases of the probiotic treatment: early (Day 0–1) **(D)**, middle (Day 4–5) **(E)**, and late (Day 8–11) **(F)**. Each network visually represents the complex interactions and correlations between different bacterial taxa, highlighting how these relationships evolve over the course of the treatment.

As treated animals showed a distinct gut microbiota structure, the microbiota profiles of individual calves were evaluated during treatment. Three claves showed distinct microbiota profiles (Figures 5A–C). Calf 2-402, with severe bloody diarrhea, showed a remarkable change in microbiota composition during probiotics treatment (Figure 5A). Initially, *Bacteroides, Escherichia-Shigella, Streptococcus, Cachnoclostridium*, and *Veillonella* were predominant on Day 0, but the relative abundance of these taxa decreased during treatment. Meanwhile, *Lactobacillus, Bifidobacterium, Megasphaera* increased. Calf 23-616 and calf 23-674, both with severe watery diarrhea, harbored different taxa compared to Calf 2-402 (Figures 5B,C). On Day 0, *Bacteroides, Escherichia-Shigella, Fusobacterium, Megasphaera, Alloprevotella, Anaerovibrio,* and *Sutterella* were predominant. Although a notable change in the morphology of calf feces was observed starting from day 1 post-treatment, the relative abundance of *Lactobacillus* and *Bifidobacterium* did not increase. *Escherichia-Shigella* continued to dominate the gut microbiota of calf 23-616 from days 2 to 6 post-treatment (Figure 5B). These data indicate that calves with NCD have distinct microbiota composition among animals and respond to probiotic treatment differently.

### 3.6 Microbe-microbe interactions altered by probiotics administration

To further understand the dynamic microbe-microbe interactions, which shape the gut microbiota, throughout probiotic treatment, we conducted a co-occurrence network analysis (Wei et al., 2020). In the early stages of treatment (Day 0-1), a dense network of both positive (*n* = 545) and negative (*n* = 260) interactions was observed, suggesting a complex and unstable microbial community due to diarrhea (Figure 5D). Nonetheless, we observed significant positive correlations between the potential diarrheagenic agent *Escherichia-Shigella* and *Erysipelatoclostridium*, *Staphylococcus*, and *Clostridium_sensu_stricto_18*. On days 4–5 post-treatment, the network appeared less dense than Day 0, comprising 235 edges, including 121 positive and 114 negative co-occurrences among 44 bacterial genera (Figure 5E), indicating changes in microbial interactions as the treatment progressed. Notably, *Lactobacillus* exhibited a significant negative correlation with eight bacterial taxa while showing positive correlations with three taxa, including *[Ruminococcus]_gnavus_group*, *Clostridium_sensu_stricto_1, Veillonella*, and *Enterococcus*; *Escherichia-Shigella* displayed positive associations with *Alloprevotella*, *UCG-005*, and *Prevotella*. On days 8–11 post-treatment, the network showed fewer edges, including positive connections (*n* = 60) and negative connections (*n* = 31) (Figure 5F). We identified significant negative correlations were identified between *Lactobacillus* and other bacteria, including *Colidextribacter*, *Butyricimonas*, *Desulfovibrio*, *Pseudoflavonifractor*, and *UBA1819*. This co-occurrence network analysis suggests that the probiotic treatment might enhance gut microbiota homeostasis, with beneficial taxa establishing stronger positive interactions and potentially detrimental taxa being suppressed or their influence reduced.

### 3.7 Identification of health-related bacterial taxa and predicted improved health index during probiotic treatment using machine learning

**Figure 6:**
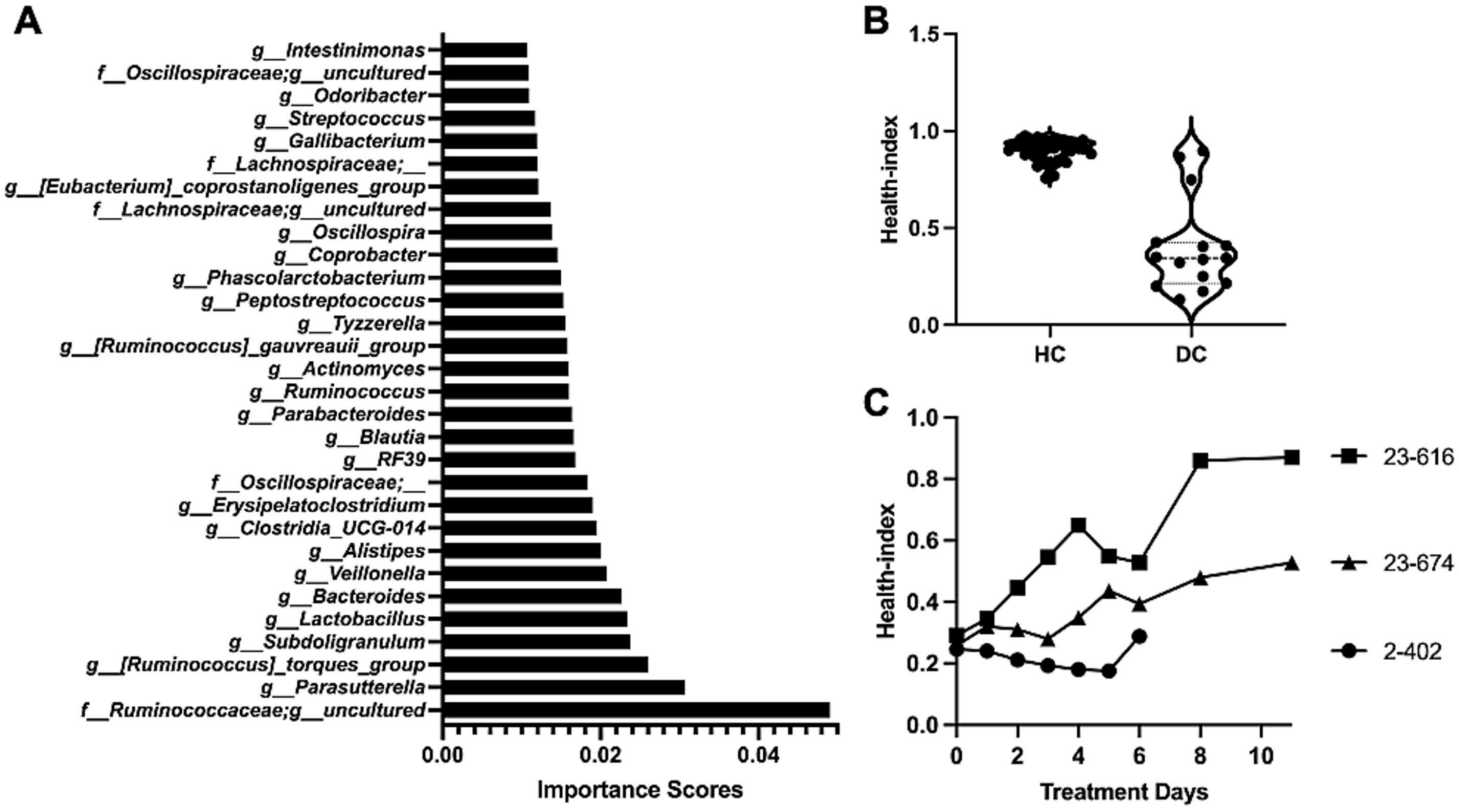
Random forest (RF) model assisted health index prediction. **(A)** Ranking of health-associated bacterial taxa. The importance scores of input taxa were evaluated by the RF model, with the top 30 important taxa presented. **(B)** Health index predictions by the RF model. Healthy calves (HC) typically exhibited a health index of around 1, while diarrheic calves (DC) primarily showed a Health Index below 0.5. **(C)** Changes of health index during *in vivo* treatment. The microbiota data was utilized in the RF model, and the health index was indicated for each day throughout the treatment course. Overall, the health index scores of all three calves with probiotic treatment demonstrated progressive improvement in gut health status over the observed period.

To assess the health status of the gut microbiota and identify key taxa that are associated gut health, we implemented and used a machine learning model in the Scikit-learn package. In particular, we trained a random forest classifier on data consisting of the relative abundance of the bacterial taxa and health status of calves. The former consists of the features for the model and the latter is the label of the data. Three-fold cross-validation was used to evaluate the classifier. We divided the data into three equal parts. In each of the three steps, one part was used to test, and the other two parts were for training our models and ranking features. Next, we identified the specific bacterial taxa that are most-associated to health-status by calculated the “importance score” of each taxon in the trained classifier, where the importance score is used to evaluate the significance of each feature towards the prediction of the model. These scores help in understanding which features are most influential in predicting the target variable, and they can significantly impact model interpretation and feature selection processes. In Random Forrest classifiers in sklearn, the importance score is given for each feature of the data, with the sum of all feature importance scores equal to one. The importance of a feature is computed as the (normalized) total reduction of the criterion brought by that feature, which is also known as Gini importance or mean decrease in impurity. We give the importance score of each feature in Supplementary Table S5. The top 30 bacterial taxa with a high importance value are highlighted in Figure 6A, with *Ruminococceae*, *Parasutterella, Ruminococcus torques*, *Subdoligranulum, and Lactobacillus* identified as the top five significant taxa of calf health status.

Finally, to optimize our model, we retrained our model on all taxa (features) with an importance score greater than zero. Our final model achieved a mean accuracy score of 86.49% (std dev 0.03), demonstrating the capability of the model to accurately predict the health status of calves based on their microbiota profiles. The calves that shed normal feces (HC) were predicted to have a health index close to one, while calves with diarrhea (DC) predominantly displayed less than 0.5 (Figure 6B). Subsequently, the health index was applied to predict the gut health status of three calves treated with probiotics. All three calves exhibited an improvement in health status (Figure 6C). Notably, calf 23-616, initially scoring below 0.4, showed its health index increased to 0.9 following probiotic treatment. Although the improvements in calf 2-402 and 23-674 were less effective, their health index exhibited increasing trends, indicating probiotic treatment was effective. Taken together, the ML model not only effectively predicted the health status of calves based on their gut microbiome but also provided valuable insights into the specific bacterial taxa contributing to the microbiome restructuring during probiotic treatment.

## 4 Discussion

In this study, we developed potential probiotics for the treatment of NCD. Orally administered *L. reuteri* strains isolated from healthy newborn calves restored the gut microbiota and relieved diarrhea. Additionally, we identified bacterial taxa associated with gut health and developed a health index for calves with diarrhea.

Previous studies have shown that probiotics play a pivotal role in improving gut health, reducing the incidence of diarrhea, and promoting growth in preweaning calves (Cangiano et al., 2020; Liu et al., 2022; Wang et al., 2023). Bacterial species, such as *Lactobacillus*, *Bifidobacterium*, *Bacillus*, and *Enterococcus,* are commonly used in probiotics. These probiotics establish a stable and nutrient-rich gut environment, effectively curbing the infiltration of pathogens and enhancing both digestive efficiency and the mucosal immune response (Collado et al., 2012; Ghazisaeedi et al., 2022; Li et al., 2023; Mills et al., 2023; Sharma et al., 2023; Sun et al., 2020; Tachibana et al., 2020). However, it is worth noting that the heterogeneity observed in the outcomes of probiotic supplementation (Alawneh et al., 2020; Cangiano et al., 2020; USDA, 2011; Xiang et al., 2023). Interestingly, the most pronounced positive effects of probiotics tend to manifest when calves are experiencing high levels of stress and disease incidence; conversely, under relatively normal or less challenging conditions, the effects of probiotic supplementation may not reach statistical significance (USDA, 2011). Furthermore, younger calves tend to exhibit a greater diversity of responses to probiotics (Alawneh et al., 2020). This suggests that the age and developmental stage of the calf may significantly influence the efficacy of probiotic supplementation. These findings underscored the complexity of the probiotic landscape and the need for tailored approaches in probiotic therapy.

Calf 2-402, which presented severe bloody diarrhea (Figure 4A), was treated with probiotics effectively. The relative abundance of potential diarrheagenic taxa associated with gastrointestinal disorders or causing severe diarrhea was decreased, but the beneficial bacterial genera *Lactobacillus* and *Bifidobacterium* were increased (Figure 5A), which are known for their positive role in gut health and immune modulation (Presti et al., 2015). These shifts in microbial populations post-probiotic treatment not only indicate a restoration of microbial balance but also suggest a potential mechanism through which the probiotic treatment mitigates diarrheal symptoms and promotes gut health. The findings underscore the therapeutic potential of probiotics in cases of severe gastrointestinal disturbances in calves. In calf 23-616 and calf 23-674, we did not detect increased prevalence of *Lactobacillus* and *Bifidobacterium* but *Escherichia-Shigella*, Bacteroides, and *Fusobacterium* were decreased by treatment. *Fusobacterium* is associated with severe watery feces and *Fusobacterium nucleatum* can secrete outer membrane vesicles that promote intestinal inflammation (Fan P. et al., 2021), potentially contributing to the occurrence of diarrhea (Engevik et al., 2021). These microbiome changes suggest that the tested probiotic strains may significantly contribute to increasing beneficial bacteria and reducing diarrhea-associated bacteria in the calf’s gut, thereby aiding in the treatment of calf diarrhea. Collectively, the probiotic treatment fostered an increase in microbial richness and induced personalized shifts in the gut microbial composition. These findings support that native-originated probiotic treatments could be pivotal for customized animal health management strategies.

Probiotics can improve animal health when probiotic strains are able to survive gastrointestinal condition and colonize the epithelium (Madsen et al., 2001; Ohland and MacNaughton, 2010; Shi et al., 2014). Tested *L. reuteri* strains not only showed enhanced viability in gastrointestinal simulations (Figure 3), but also carried genes associated with stresses such as temperature, pH, bile salts, and oxidation (Figure 1C; Supplementary Table S2). Diverse genes associated with these stresses are necessary to survive pass through the GI tract. Furthermore, bacteriocin enterolysin A can suppress pathogenic bacteria in the GI tract and enhance animal health through the inhibition of bacterial growth by cell wall lysis (Anjana and Tiwari, 2022; Nilsen et al., 2003). Furthermore, the safety of probiotics was evaluated as they might produce adverse effects on host health by encoding antibiotic resistance genes (Castro-López et al., 2021). However, any functional antibiotic resistance genes were identified by *in silico* analysis.

As *in silico* analysis continues to evolve, ML technology has emerged as an indispensable tool, significantly contributing to the precision understanding of probiotics. Particularly notable is its application in the realm of gut microbiome research, where ML models prove instrumental in handling large datasets, unraveling complex patterns, and predicting the effectiveness of interventions targeting gut microbes, alongside personalized healthcare solutions (Das et al., 2023; Li et al., 2022; Liu et al., 2022; Tan et al., 2023). Westfall et al. employed a multivariate adaptive regression splines model to formulate probiotics tailored to specific therapeutic properties, guided by the distinctive metabolic activities of these microorganisms (Westfall et al., 2021). Sun et al. introduced a ML platform named iProbiotics, which utilized a support vector machine algorithm to discern probiotic characteristics from whole-genome sequencing data (Sun et al., 2022). Furthermore, McCoubrey et al. applied a ML model based on a random forest classifier to select functional excipients optimizing probiotic growth in the gastrointestinal tract (McCoubrey et al., 2022). By recognizing the potential of the gut microbiome as a marker of host health, Gupta et al. introduced the Gut Microbiome Health Index (GMHI), a biologically interpretable mathematical formula designed to predict disease likelihood independently of clinical diagnoses (Gupta et al., 2020). In this study, we employed a ML model to predict the health status of calves based on their microbiome profiles. This approach allowed for a comprehensive assessment of the effects of *L. reuteri* on calf health. Compared to traditional statistical methods, this ML-based approach offered a more comprehensive view, encompassing various health indicators and bacterial species. The results not only validated our microbiome findings but also enhanced our understanding of the effects of probiotic strains. Although these studies underscored the critical role of ML in probiotics research, they also emphasized the need for further phenotypic validation for probiotic selection and refined modeling techniques.

This study has provided valuable insights into the prospect of harnessing the natural gut microbiota for probiotic development. While these findings are promising, the long-term effects of probiotic treatment on gut health and overall calf performance need to be explored. Additionally, further studies are required to understand the mechanism of action of *L. reuteri* in different environmental and dietary conditions with more animals.

## 5 Conclusion

NCD is a major cause of calf deaths, leading to widespread antibiotic use and concerns about antimicrobial resistance. We screened potential probiotics *L. reuteri* using WGS, *in silico*, and *in vitro* analyses, and then *L. reuteri strains* were administered to diarrheic calves. Potential probiotics *L. reuteri* improved diarrhea and animal behavior. Machine learning analysis identified beneficial bacterial taxa and predicted the health status of calves, marking progress in antibiotic alternatives. In conclusion, this study adds to the growing body of evidence that supports the use of native gut microbiota-derived probiotics in veterinary practice, aiding global efforts in mitigating antimicrobial resistance and promoting animal health and welfare.

## Data Availability

The datasets presented in this study can be found in online repositories. The names of the repository/repositories and accession number(s) can be found in the article/Supplementary material.

## Footnotes

^1^ https://www.arb-silva.de/documentation/release-132/

^2^ https://seaborn.pydata.org/

^3^ www.graphpad.com
