# Supplementary material for: Machine learning-enhanced assessment of potential probiotics from healthy calves for the treatment of neonatal calf diarrhea
Source: Front Microbiol. 2024 Dec 9;15:1507537. doi: 10.3389/fmicb.2024.1507537 (PMC11663915; doi:10.3389/fmicb.2024.1507537)
Supplement: Supplementary file 1 [file Table_1.docx]

Supplementary Material

**Machine learning-enhanced Assessment of potential probiotics from Healthy Calves for the Treatment of Neonatal Calf Diarrhea**

**Yuting Zhai^1,2^**^,^**^†^, Miju Kim^1,2,3,†^ , Peixin Fan^1,+^, Sharath Rajeev^2^, Sun Ae Kim^4^, J. Danny Driver^1^, Klibs N. Galvão^5^, Christina Boucher^6^, and Kwangcheol C. Jeong^1,2*^**

^1^Department of Animal Sciences, University of Florida, Gainesville, FL, USA

^2^ Emerging Pathogens Institute, University of Florida, Gainesville, FL, USA

^3^Department of Food Science and Biotechnology, Kyung Hee University, Seoul, ROK

^4^Department of Food Science and Biotechnology, Ewha Womans University, Seoul, ROK

^5^Department of Large Animal Clinical Sciences, University of Florida, Gainesville, USA

^6^Department of Computer and Information Science and Engineering, University of Florida, Gainesville, FL, USA

*** Correspondence:**Kwangcheol Casey Jeong, PhD

kcjeong@ufl.edu

^† :^ These authors contributed equally

^+^: Currently at Mississippi State University

**Supplementary Tables**

**Supplementary Table 1. *L. reuteri* strains from this study.**

| **Strain** | **Sample accession** | **Genome accession** |
| --- | --- | --- |
| KCJ2K2606 | SAMN38912182 | JAYFHM000000000 |
| KCJ2K2610 | SAMN38912183 | JAYFHN000000000 |
| KCJ2K2614 | SAMN38912184 | JAYFHO000000000 |
| KCJ2K2615 | SAMN38912185 | JAYFHP000000000 |
| KCJ2K2620 | SAMN38912186 | JAYFHQ000000000 |
| KCJ2K2623 | SAMN38912187 | JAYFHR000000000 |
| KCJ2K2624 | SAMN38912188 | JAYFHS000000000 |
| KCJ2K2628 | SAMN38912189 | JAYFHT000000000 |
| KCJ2K2631 | SAMN38912190 | JAYFHU000000000 |
| KCJ2K2638 | SAMN38912191 | JAYFHV000000000 |
| KCJ2K2639 | SAMN38912192 | JAYFHW000000000 |
| KCJ2K2641 | SAMN38912193 | JAYFHX000000000 |
| KCJ2K2646 | SAMN38912194 | JAYFHY000000000 |
| KCJ2K2647 | SAMN38912195 | JAYFHZ000000000 |
| KCJ2K2653 | SAMN38912196 | JAYFIA000000000 |
| KCJ2K2660 | SAMN38912197 | JAYFIB000000000 |
| KCJ2K2663 | SAMN38912198 | JAYFIC000000000 |
| KCJ2K2673 | SAMN38912199 | JAYFID000000000 |
| KCJ2K2674 | SAMN38912200 | JAYFIE000000000 |
| KCJ2K2679 | SAMN38912201 | JAYFIF000000000 |
| KCJ2K2681 | SAMN38912202 | JAYFIG000000000 |
| KCJ2K2682 | SAMN38912203 | JAYFIH000000000 |

**Supplementary Table 2. Genes associated with probiotic function**

| **Function** | **Strains** | | | | | | | | |
| --- | --- | --- | --- | --- | --- | --- | --- | --- | --- |
|  | KCJ2K2614 | KCJ2K2615 | KCJ2K2639 | KCJ2K2646 | KCJ2K2653 | KCJ2K2660 | KCJ2K2663 | KCJ2K2673 | KCJ2K2674 |
| **Bacteriocin** | | | | | | | | | |
| *enlA* | + | + | + | + | + | + | + | + | + |
| **Temperature** | | | | | | | | | |
| *cspB* | + | + | + | + | + | + | + | + | + |
| *cspC* | + | + | + | + | + | + | + | + | + |
| *dnaK* | + | + | + | + | + | + | + | + | + |
| *grpE* | + | + | + | + | + | + | + | + | + |
| *hsp* | *+* | *-* | *+* | *+* | *+* | *+* | *+* | *+* | *+* |
| **pH** | | | | | | | | | |
| *itaS* | + | + | + | + | + | + | + | + | + |
| *atpH* | + | + | + | + | + | + | + | + | + |
| *atpE* | + | + | + | + | + | + | + | + | + |
| *nhak* | + | + | + | + | + | + | + | + | + |
| *nhaP3* | *+* | *+* | *+* | *+* | *+* | *+* | *+* | *+* | *+* |
| **Bile resistance** | | | | | | | | | |
| *cbh* | + | + | + | + | + | + | + | + | + |
| *ppaC* | + | + | + | + | + | + | + | + | + |
| *glpK* | + | + | + | + | + | + | + | + | + |
| *srtA* | *+* | *+* | *+* | *+* | *+* | *+* | *+* | *+* | *+* |
| **Oxidation** | | | | | | | | | |
| *nox* | + | + | + | + | + | + | + | + | + |
| *yumB* | + | + | + | + | + | + | + | + | + |

**Supplementary Table 2. Continues**

| **Function** | **Strains** | | | | | | | | |
| --- | --- | --- | --- | --- | --- | --- | --- | --- | --- |
|  | KCJ2K2614 | KCJ2K2615 | KCJ2K2639 | KCJ2K2646 | KCJ2K2653 | KCJ2K2660 | KCJ2K2663 | KCJ2K2673 | KCJ2K2674 |
| *iolU* | + | + | + | + | + | + | + | + | + |
| *usp5* | + | + | + | + | + | + | + | + | + |
| *uspA* | + | + | + | + | + | + | + | + | + |
| *uspA3* | + | + | + | + | + | + | + | + | + |
| *ytpP* | + | + | + | + | + | + | + | + | + |
| *yjbH* | + | + | + | + | + | + | + | + | + |

**Supplementary Table 3**

| **Strain** | **ARO term** | **% Length of Reference Sequence** | **% Identify of Matching Region** |
| --- | --- | --- | --- |
| KCJ2K2614 | *vanT* gene in *vanG* cluster | 52.67 | 34.49 |
| KCJ2K2615 | *vanT* gene in *vanG* cluster | 52.67 | 34.49 |
| KCJ2K2639 | *vanT* gene in *vanG* cluster | 52.67 | 34.49 |
| KCJ2K2646 | *vanT* gene in *vanG* cluster | 52.67 | 34.49 |
| KCJ2K2653 | *vanT* gene in *vanG* cluster | 52.67 | 34.49 |
| KCJ2K2660 | *vanT* gene in *vanG* cluster | 52.67 | 34.49 |
| KCJ2K2663 | *vanT* gene in *vanG* cluster | 52.67 | 34.49 |
| KCJ2K2673 | *vanT* gene in *vanG* cluster | 52.67 | 34.49 |
| KCJ2K2674 | *vanT* gene in *vanG* cluster | 52.67 | 34.49 |

**Supplementary Table 4. Strains from NCBI**

| **Labels** | **Hosts** | **NCBI Accession** |
| --- | --- | --- |
| GCF_003175075.1_ASM317507v1_genomic | Cow | GCF_003175075.1 |
| GCF_003175035.1_ASM317503v1_genomic | Cow | GCF_003175035.1 |
| GCF_003174945.1_ASM317494v1_genomic | Cow | GCF_003174945.1 |
| GCF_003174855.1_ASM317485v1_genomic | Cow | GCF_003174855.1 |
| GCA_002160565.1_ASM216056v1_genomic | Chicken | GCA_002160565.1 |
| GCA_002159305.1_ASM215930v1_genomic | Chicken | GCA_002159305.1 |
| GCA_002112245.1_ASM211224v1_genomic | Chicken | GCA_002112245.1 |
| GCA_002112225.1_ASM211222v1_genomic | Chicken | GCA_002112225.1 |
| GCA_002112185.1_ASM211218v1_genomic | Chicken | GCA_002112185.1 |
| GCF_003175125.1_ASM317512v1_genomic | Goat | GCF_003175125.1 |
| GCF_003175015.1_ASM317501v1_genomic | Goat | GCF_003175015.1 |
| GCF_003174995.1_ASM317499v1_genomic | Goat | GCF_003174995.1 |
| GCF_003174955.1_ASM317495v1_genomic | Goat | GCF_003174955.1 |
| GCF_003174935.1_ASM317493v1_genomic | Goat | GCF_003174935.1 |
| GCF_024652885.1_ASM2465288v1_genomic | Human | GCF_024652885.1 |
| GCA_021398615.1_ASM2139861v1_genomic | Human | GCA_021398615.1 |
| GCA_020023775.1_ASM2002377v1_genomic | Human | GCA_020023775.1 |
| GCA_002112805.1_ASM211280v1_genomic | Human | GCA_002112805.1 |
| GCA_002112195.1_ASM211219v1_genomic | Human | GCA_002112195.1 |
| GCF_013487925.1_ASM1348792v1_genomic | Horse | GCF_013487925.1 |
| GCF_003175025.1_ASM317502v1_genomic | Horse | GCF_003175025.1 |
| GCF_003174915.1_ASM317491v1_genomic | Horse | GCF_003174915.1 |
| GCF_003174815.1_ASM317481v1_genomic | Horse | GCF_003174815.1 |
| GCA_002254255.1_ASM225425v1_genomic | Mouse | GCA_002254255.1 |
| GCA_002254245.1_ASM225424v1_genomic | Mouse | GCA_002254245.1 |
| GCA_002254195.1_ASM225419v1_genomic | Mouse | GCA_002254195.1 |
| GCA_002254185.1_ASM225418v1_genomic | Mouse | GCA_002254185.1 |

**Supplementary Table 4. Continue**

| **Labels** | **Hosts** | **NCBI Accession** |
| --- | --- | --- |
| GCA_002254175.1_ASM225417v1_genomic | Mouse | GCA_002254175.1 |
| GCA_002254165.1_ASM225416v1_genomic | Mouse | GCA_002254165.1 |
| GCA_002254115.1_ASM225411v1_genomic | Mouse | GCA_002254115.1 |
| GCA_002254105.1_ASM225410v1_genomic | Mouse | GCA_002254105.1 |
| GCA_002254095.1_ASM225409v1_genomic | Mouse | GCA_002254095.1 |
| GCA_002254085.1_ASM225408v1_genomic | Mouse | GCA_002254085.1 |
| GCA_002254045.1_ASM225404v1_genomic | Mouse | GCA_002254045.1 |
| GCA_002254035.1_ASM225403v1_genomic | Mouse | GCA_002254035.1 |
| GCA_002254025.1_ASM225402v1_genomic | Mouse | GCA_002254025.1 |
| GCA_002253975.1_ASM225397v1_genomic | Mouse | GCA_002253975.1 |
| GCA_002253965.1_ASM225396v1_genomic | Mouse | GCA_002253965.1 |
| GCA_002253955.1_ASM225395v1_genomic | Mouse | GCA_002253955.1 |
| GCA_002253945.1_ASM225394v1_genomic | Mouse | GCA_002253945.1 |
| GCA_002253925.1_ASM225392v1_genomic | Mouse | GCA_002253925.1 |
| GCA_002253905.1_ASM225390v1_genomic | Mouse | GCA_002253905.1 |
| GCA_002253875.1_ASM225387v1_genomic | Mouse | GCA_002253875.1 |
| GCA_002253865.1_ASM225386v1_genomic | Mouse | GCA_002253865.1 |
| GCA_002253835.1_ASM225383v1_genomic | Mouse | GCA_002253835.1 |
| GCA_002253825.1_ASM225382v1_genomic | Mouse | GCA_002253825.1 |
| GCA_002253785.1_ASM225378v1_genomic | Mouse | GCA_002253785.1 |
| GCA_002253765.1_ASM225376v1_genomic | Mouse | GCA_002253765.1 |
| GCA_002253755.1_ASM225375v1_genomic | Mouse | GCA_002253755.1 |
| GCA_002253745.1_ASM225374v1_genomic | Mouse | GCA_002253745.1 |
| GCA_002253725.1_ASM225372v1_genomic | Mouse | GCA_002253725.1 |
| GCA_002253705.1_ASM225370v1_genomic | Mouse | GCA_002253705.1 |
| GCA_002253685.1_ASM225368v1_genomic | Mouse | GCA_002253685.1 |
| GCA_002253665.1_ASM225366v1_genomic | Mouse | GCA_002253665.1 |

**Supplementary Table 4. Continue**

| **Labels** | **Hosts** | **NCBI Accession** |
| --- | --- | --- |
| GCA_002253625.1_ASM225362v1_genomic | Mouse | GCA_002253625.1 |
| GCA_002221655.1_ASM222165v1_genomic | Mouse | GCA_002221655.1 |
| GCA_002156605.1_ASM215660v1_genomic | Mouse | GCA_002156605.1 |
| GCA_002888655.1_ASM288865v1_genomic | Pig | GCA_002888655.1 |
| GCA_002128765.1_ASM212876v1_genomic | Pig | GCA_002128765.1 |
| GCA_002128755.1_ASM212875v1_genomic | Pig | GCA_002128755.1 |
| GCA_002128745.1_ASM212874v1_genomic | Pig | GCA_002128745.1 |
| GCA_002128715.1_ASM212871v1_genomic | Pig | GCA_002128715.1 |
| GCA_002128705.1_ASM212870v1_genomic | Pig | GCA_002128705.1 |
| GCA_002128685.1_ASM212868v1_genomic | Pig | GCA_002128685.1 |
| GCA_002128655.1_ASM212865v1_genomic | Pig | GCA_002128655.1 |
| GCA_002128635.1_ASM212863v1_genomic | Pig | GCA_002128635.1 |
| GCA_002128615.1_ASM212861v1_genomic | Pig | GCA_002128615.1 |
| GCA_002128605.1_ASM212860v1_genomic | Pig | GCA_002128605.1 |
| GCA_002128585.1_ASM212858v1_genomic | Pig | GCA_002128585.1 |
| GCA_002128555.1_ASM212855v1_genomic | Pig | GCA_002128555.1 |
| GCA_002128525.1_ASM212852v1_genomic | Pig | GCA_002128525.1 |
| GCA_002128495.1_ASM212849v1_genomic | Pig | GCA_002128495.1 |
| GCA_002128485.1_ASM212848v1_genomic | Pig | GCA_002128485.1 |
| GCA_002762415.1_ASM276241v1_genomic | Probiotic product | GCA_002762415.1 |
| GCF_015377805.1_ASM1537780v1_genomic | Probiotic product | GCF_015377805.1 |
| GCF_003175115.1_ASM317511v1_genomic | Sheep | GCF_003175115.1 |
| GCF_003175085.1_ASM317508v1_genomic | Sheep | GCF_003175085.1 |
| GCF_003174875.1_ASM317487v1_genomic | Sheep | GCF_003174875.1 |
| GCF_003174865.1_ASM317486v1_genomic | Sheep | GCF_003174865.1 |
| GCA_003046135.1_ASM304613v1_genomic | Sourdough | GCA_003046135.1 |
| GCA_003046055.1_ASM304605v1_genomic | Sourdough | GCA_003046055.1 |

**Supplementary Table 5. ML model identified health-related bacterial taxa**

| **Taxa** | **Importance** |
| --- | --- |
| d__Bacteria;p__Firmicutes;c__Clostridia;o__Oscillospirales;f__Ruminococcaceae;g__uncultured | 0.04904 |
| d__Bacteria;p__Proteobacteria;c__Gammaproteobacteria;o__Burkholderiales;f__Sutterellaceae;g__Parasutterella | 0.030664 |
| d__Bacteria;p__Firmicutes;c__Clostridia;o__Lachnospirales;f__Lachnospiraceae;g__[Ruminococcus]_torques_group | 0.026039 |
| d__Bacteria;p__Firmicutes;c__Clostridia;o__Oscillospirales;f__Ruminococcaceae;g__Subdoligranulum | 0.02378 |
| d__Bacteria;p__Firmicutes;c__Bacilli;o__Lactobacillales;f__Lactobacillaceae;g__Lactobacillus | 0.023364 |
| d__Bacteria;p__Bacteroidota;c__Bacteroidia;o__Bacteroidales;f__Bacteroidaceae;g__Bacteroides | 0.022661 |
| d__Bacteria;p__Firmicutes;c__Negativicutes;o__Veillonellales-Selenomonadales;f__Veillonellaceae;g__Veillonella | 0.020744 |
| d__Bacteria;p__Bacteroidota;c__Bacteroidia;o__Bacteroidales;f__Rikenellaceae;g__Alistipes | 0.02002 |
| d__Bacteria;p__Firmicutes;c__Clostridia;o__Clostridia_UCG-014;f__Clostridia_UCG-014;g__Clostridia_UCG-014 | 0.019513 |
| d__Bacteria;p__Firmicutes;c__Bacilli;o__Erysipelotrichales;f__Erysipelatoclostridiaceae;g__Erysipelatoclostridium | 0.019 |
| d__Bacteria;p__Firmicutes;c__Clostridia;o__Oscillospirales;f__Oscillospiraceae;__ | 0.018314 |
| d__Bacteria;p__Firmicutes;c__Bacilli;o__RF39;f__RF39;g__RF39 | 0.01676 |
| d__Bacteria;p__Firmicutes;c__Clostridia;o__Lachnospirales;f__Lachnospiraceae;g__Blautia | 0.016572 |
| d__Bacteria;p__Bacteroidota;c__Bacteroidia;o__Bacteroidales;f__Tannerellaceae;g__Parabacteroides | 0.01639 |
| d__Bacteria;p__Firmicutes;c__Clostridia;o__Oscillospirales;f__Ruminococcaceae;g__Ruminococcus | 0.015982 |
| d__Bacteria;p__Actinobacteriota;c__Actinobacteria;o__Actinomycetales;f__Actinomycetaceae;g__Actinomyces | 0.015972 |
| d__Bacteria;p__Firmicutes;c__Clostridia;o__Lachnospirales;f__Lachnospiraceae;g__[Ruminococcus]_gauvreauii_group | 0.015782 |
| d__Bacteria;p__Firmicutes;c__Clostridia;o__Lachnospirales;f__Lachnospiraceae;g__Tyzzerella | 0.015514 |
| d__Bacteria;p__Firmicutes;c__Clostridia;o__Peptostreptococcales-Tissierellales;f__Peptostreptococcaceae;g__Peptostreptococcus | 0.015277 |
| d__Bacteria;p__Firmicutes;c__Negativicutes;o__Acidaminococcales;f__Acidaminococcaceae;g__Phascolarctobacterium | 0.01496 |
| d__Bacteria;p__Bacteroidota;c__Bacteroidia;o__Bacteroidales;f__Barnesiellaceae;g__Coprobacter | 0.014505 |
| d__Bacteria;p__Firmicutes;c__Clostridia;o__Oscillospirales;f__Oscillospiraceae;g__Oscillospira | 0.01381 |

**Supplementary Table 5. Continue**

| **Taxa** | **Importance** |
| --- | --- |
| d__Bacteria;p__Firmicutes;c__Clostridia;o__Lachnospirales;f__Lachnospiraceae;g__uncultured | 0.013704 |
| d__Bacteria;p__Firmicutes;c__Clostridia;o__Oscillospirales;f__[Eubacterium]_coprostanoligenes_group;g__[Eubacterium]_coprostanoligenes_group | 0.012099 |
| d__Bacteria;p__Firmicutes;c__Clostridia;o__Lachnospirales;f__Lachnospiraceae;__ | 0.012038 |
| d__Bacteria;p__Proteobacteria;c__Gammaproteobacteria;o__Pasteurellales;f__Pasteurellaceae;g__Gallibacterium | 0.011947 |
| d__Bacteria;p__Firmicutes;c__Bacilli;o__Lactobacillales;f__Streptococcaceae;g__Streptococcus | 0.011683 |
| d__Bacteria;p__Bacteroidota;c__Bacteroidia;o__Bacteroidales;f__Marinifilaceae;g__Odoribacter | 0.01096 |
| d__Bacteria;p__Firmicutes;c__Clostridia;o__Oscillospirales;f__Oscillospiraceae;g__uncultured | 0.010891 |
| d__Bacteria;p__Firmicutes;c__Clostridia;o__Oscillospirales;f__Oscillospiraceae;g__Intestinimonas | 0.010707 |
| d__Bacteria;p__Desulfobacterota;c__Desulfovibrionia;o__Desulfovibrionales;f__Desulfovibrionaceae;g__Bilophila | 0.010351 |
| d__Bacteria;p__Firmicutes;c__Clostridia;o__Lachnospirales;f__Lachnospiraceae;g__Lachnospiraceae_UCG-004 | 0.010086 |
| d__Bacteria;p__Firmicutes;c__Clostridia;o__Oscillospirales;f__Ruminococcaceae;g__Anaerofilum | 0.009913 |
| d__Bacteria;p__Bacteroidota;c__Bacteroidia;o__Bacteroidales;f__Prevotellaceae;g__Prevotella | 0.009852 |
| d__Bacteria;p__Firmicutes;c__Bacilli;o__Lactobacillales;f__Enterococcaceae;g__Enterococcus | 0.009582 |
| d__Bacteria;p__Firmicutes;c__Clostridia;o__Oscillospirales;f__Oscillospiraceae;g__Colidextribacter | 0.009546 |
| d__Bacteria;p__Firmicutes;c__Clostridia;o__Christensenellales;f__Christensenellaceae;g__Christensenellaceae_R-7_group | 0.00954 |
| d__Bacteria;p__Firmicutes;c__Clostridia;o__Lachnospirales;f__Lachnospiraceae;g__Coprococcus | 0.009383 |
| d__Bacteria;p__Firmicutes;c__Clostridia;o__Oscillospirales;f__Ruminococcaceae;g__Negativibacillus | 0.009289 |
| d__Bacteria;p__Firmicutes;c__Clostridia;o__Lachnospirales;f__Lachnospiraceae;g__Lachnospiraceae_AC2044_group | 0.009186 |
| d__Bacteria;p__Firmicutes;c__Clostridia;o__Oscillospirales;f__Ruminococcaceae;g__UBA1819 | 0.009129 |
| d__Bacteria;p__Bacteroidota;c__Bacteroidia;o__Bacteroidales;f__Marinifilaceae;g__Butyricimonas | 0.008961 |
| d__Bacteria;p__Firmicutes;c__Clostridia;o__Oscillospirales;f__Oscillospiraceae;g__Oscillibacter | 0.008698 |
| d__Bacteria;p__Firmicutes;c__Clostridia;o__Oscillospirales;f__Ruminococcaceae;g__Faecalibacterium | 0.008606 |
| d__Bacteria;p__Actinobacteriota;c__Coriobacteriia;o__Coriobacteriales;f__Coriobacteriaceae;g__Collinsella | 0.008021 |

**Supplementary Table 5. Continue**

| **Taxa** | **Importance** |
| --- | --- |
| d__Bacteria;p__Firmicutes;c__Clostridia;o__Oscillospirales;f__Butyricicoccaceae;g__Butyricicoccus | 0.007677 |
| d__Bacteria;p__Firmicutes;c__Clostridia;o__Lachnospirales;f__Lachnospiraceae;g__Dorea | 0.007452 |
| d__Bacteria;p__Bacteroidota;c__Bacteroidia;o__Bacteroidales;f__uncultured;g__uncultured | 0.007398 |
| d__Bacteria;p__Firmicutes;c__Clostridia;o__Lachnospirales;f__Lachnospiraceae;g__Lachnospiraceae_UCG-010 | 0.007311 |
| d__Bacteria;p__Firmicutes;c__Clostridia;o__Lachnospirales;f__Lachnospiraceae;g__Sellimonas | 0.007304 |
| d__Bacteria;p__Fusobacteriota;c__Fusobacteriia;o__Fusobacteriales;f__Fusobacteriaceae;g__Fusobacterium | 0.007116 |
| d__Bacteria;p__Firmicutes;c__Clostridia;o__Lachnospirales;f__Lachnospiraceae;g__Lachnospiraceae_NK4A136_group | 0.007019 |
| d__Bacteria;p__Firmicutes;c__Clostridia;o__Lachnospirales;f__Lachnospiraceae;g__CAG-56 | 0.006828 |
| d__Bacteria;p__Firmicutes;c__Clostridia;o__Oscillospirales;f__Ruminococcaceae;__ | 0.006733 |
| d__Bacteria;p__Proteobacteria;c__Gammaproteobacteria;o__Enterobacterales;f__Enterobacteriaceae;g__Escherichia-Shigella | 0.006584 |
| d__Bacteria;p__Firmicutes;c__Clostridia;o__Oscillospirales;f__Oscillospiraceae;g__UCG-005 | 0.006578 |
| d__Bacteria;p__Firmicutes;c__Clostridia;o__Lachnospirales;f__Lachnospiraceae;g__Lachnoclostridium | 0.006466 |
| d__Bacteria;p__Firmicutes;c__Negativicutes;o__Veillonellales-Selenomonadales;f__Veillonellaceae;g__Megasphaera | 0.006427 |
| d__Bacteria;p__Firmicutes;c__Clostridia;o__Peptostreptococcales-Tissierellales;f__Anaerovoracaceae;g__[Eubacterium]_nodatum_group | 0.006381 |
| d__Bacteria;p__Cyanobacteria;c__Vampirivibrionia;o__Gastranaerophilales;f__Gastranaerophilales;g__Gastranaerophilales | 0.006328 |
| d__Bacteria;p__Verrucomicrobiota;c__Verrucomicrobiae;o__Verrucomicrobiales;f__Akkermansiaceae;g__Akkermansia | 0.006315 |
| d__Bacteria;p__Firmicutes;c__Clostridia;o__Clostridiales;f__Clostridiaceae;g__Clostridium_sensu_stricto_1 | 0.006101 |
| d__Bacteria;p__Firmicutes;c__Clostridia;o__Lachnospirales;f__Lachnospiraceae;g__Roseburia | 0.00574 |
| d__Bacteria;p__Proteobacteria;c__Alphaproteobacteria;o__Rhodospirillales;f__uncultured;g__uncultured | 0.005692 |
| d__Bacteria;p__Firmicutes;c__Clostridia;o__Oscillospirales;f__Oscillospiraceae;g__Pseudoflavonifractor | 0.00564 |
| d__Bacteria;p__Firmicutes;c__Clostridia;o__Oscillospirales;f__Oscillospiraceae;g__NK4A214_group | 0.0056 |
| d__Bacteria;p__Actinobacteriota;c__Coriobacteriia;o__Coriobacteriales;f__Atopobiaceae;g__Atopobium | 0.005404 |

**Supplementary Table 5. Continue**

| **Taxa** | **Importance** |
| --- | --- |
| d__Bacteria;p__Firmicutes;c__Clostridia;o__Clostridiales;f__Clostridiaceae;g__Clostridium_sensu_stricto_2 | 0.005253 |
| d__Bacteria;p__Firmicutes;c__Bacilli;o__Erysipelotrichales;f__Erysipelatoclostridiaceae;g__Erysipelotrichaceae_UCG-003 | 0.005188 |
| d__Bacteria;p__Desulfobacterota;c__Desulfovibrionia;o__Desulfovibrionales;f__Desulfovibrionaceae;g__Desulfovibrio | 0.00491 |
| d__Bacteria;p__Firmicutes;c__Clostridia;o__Oscillospirales;f__[Clostridium]_methylpentosum_group;g__[Clostridium]_methylpentosum_group | 0.004853 |
| d__Bacteria;p__Firmicutes;c__Clostridia;o__Lachnospirales;f__Lachnospiraceae;g__Lachnospiraceae_NK3A20_group | 0.004845 |
| d__Bacteria;p__Bacteroidota;c__Bacteroidia;o__Bacteroidales;f__Prevotellaceae;g__uncultured | 0.004753 |
| d__Bacteria;p__Firmicutes;c__Bacilli;o__Paenibacillales;f__Paenibacillaceae;g__Paenibacillus | 0.004685 |
| d__Bacteria;p__Firmicutes;c__Bacilli;o__Erysipelotrichales;f__Erysipelotrichaceae;g__Faecalitalea | 0.004589 |
| d__Bacteria;p__Firmicutes;c__Clostridia;o__Oscillospirales;f__Ruminococcaceae;g__Fournierella | 0.004478 |
| d__Bacteria;p__Bacteroidota;c__Bacteroidia;o__Bacteroidales;f__Barnesiellaceae;g__Barnesiella | 0.004403 |
| d__Bacteria;p__Firmicutes;c__Clostridia;o__Oscillospirales;f__Oscillospiraceae;g__Flavonifractor | 0.003983 |
| d__Bacteria;p__Spirochaetota;c__Brevinematia;o__Brevinematales;f__Brevinemataceae;g__Brevinema | 0.003957 |
| d__Bacteria;p__Firmicutes;c__Clostridia;o__Lachnospirales;f__Lachnospiraceae;g__[Acetivibrio]_ethanolgignens_group | 0.003605 |
| d__Bacteria;p__Firmicutes;c__Clostridia;o__Lachnospirales;f__Lachnospiraceae;g__[Ruminococcus]_gnavus_group | 0.003539 |
| d__Bacteria;p__Firmicutes;c__Clostridia;o__Peptococcales;f__Peptococcaceae;g__uncultured | 0.003496 |
| d__Bacteria;p__Campilobacterota;c__Campylobacteria;o__Campylobacterales;f__Campylobacteraceae;g__Campylobacter | 0.003303 |
| d__Bacteria;p__Firmicutes;c__Clostridia;o__Oscillospirales;f__Oscillospirales;g__Hydrogenoanaerobacterium | 0.003296 |
| d__Bacteria;p__Firmicutes;c__Clostridia;o__Oscillospirales;f__Oscillospiraceae;g__UCG-002 | 0.003285 |
| d__Bacteria;p__Verrucomicrobiota;c__Verrucomicrobiae;o__Chthoniobacterales;f__Chthoniobacteraceae;g__Candidatus_Udaeobacter | 0.003264 |
| d__Bacteria;p__Firmicutes;c__Bacilli;o__Erysipelotrichales;f__Erysipelotrichaceae;g__Holdemania | 0.003235 |
| d__Bacteria;p__Firmicutes;c__Clostridia;o__Lachnospirales;f__Lachnospiraceae;g__Anaerostipes | 0.003011 |
| d__Bacteria;p__Firmicutes;c__Clostridia;o__Lachnospirales;f__Lachnospiraceae;g__Frisingicoccus | 0.003011 |

**Supplementary Table 5. Continue**

| **Taxa** | **Importance** |
| --- | --- |
| d__Bacteria;p__Firmicutes;c__Clostridia;o__Oscillospirales;f__Oscillospiraceae;g__UCG-003 | 0.003001 |
| d__Bacteria;p__Firmicutes;c__Clostridia;o__Lachnospirales;f__Lachnospiraceae;g__Fusicatenibacter | 0.002935 |
| d__Bacteria;p__Firmicutes;c__Clostridia;o__Lachnospirales;f__Lachnospiraceae;g__GCA-900066575 | 0.002891 |
| d__Bacteria;p__Verrucomicrobiota;c__Lentisphaeria;o__Victivallales;f__Victivallaceae;g__Victivallis | 0.00286 |
| d__Bacteria;p__Actinobacteriota;c__Actinobacteria;o__Bifidobacteriales;f__Bifidobacteriaceae;g__Bifidobacterium | 0.002853 |
| d__Bacteria;p__Firmicutes;c__Bacilli;o__Erysipelotrichales;f__Erysipelotrichaceae;g__Turicibacter | 0.002851 |
| d__Bacteria;p__Bacteroidota;c__Bacteroidia;o__Flavobacteriales;f__Flavobacteriaceae;g__uncultured | 0.002811 |
| d__Bacteria;p__Firmicutes;c__Clostridia;o__Clostridia_vadinBB60_group;f__Clostridia_vadinBB60_group;g__Clostridia_vadinBB60_group | 0.00272 |
| d__Bacteria;p__Firmicutes;c__Bacilli;o__Erysipelotrichales;f__Erysipelatoclostridiaceae;g__UCG-004 | 0.002647 |
| d__Bacteria;p__Proteobacteria;c__Gammaproteobacteria;o__Pasteurellales;f__Pasteurellaceae;g__Actinobacillus | 0.002624 |
| d__Bacteria;p__Firmicutes;c__Negativicutes;o__Acidaminococcales;f__Acidaminococcaceae;g__Succiniclasticum | 0.002588 |
| d__Bacteria;p__Proteobacteria;c__Gammaproteobacteria;o__Pasteurellales;f__Pasteurellaceae;g__Mannheimia | 0.002575 |
| d__Bacteria;p__Bacteroidota;c__Bacteroidia;o__Bacteroidales;f__Tannerellaceae;__ | 0.002286 |
| d__Bacteria;p__Firmicutes;c__Clostridia;o__Christensenellales;f__Christensenellaceae;__ | 0.002254 |
| d__Bacteria;p__Firmicutes;c__Negativicutes;o__Veillonellales-Selenomonadales;f__Veillonellaceae;g__Allisonella | 0.002218 |
| d__Bacteria;p__Firmicutes;c__Clostridia;o__Peptostreptococcales-Tissierellales;f__Peptostreptococcaceae;g__Intestinibacter | 0.002165 |
| d__Bacteria;p__Firmicutes;c__Clostridia;o__Monoglobales;f__Monoglobaceae;g__Monoglobus | 0.002118 |
| d__Bacteria;p__Firmicutes;c__Clostridia;o__Lachnospirales;f__Lachnospiraceae;g__Lachnospira | 0.002056 |
| d__Bacteria;p__Firmicutes;c__Bacilli;o__Acholeplasmatales;f__Acholeplasmataceae;g__EMP-G18 | 0.00189 |
| d__Bacteria;p__Firmicutes;c__Clostridia;o__Peptococcales;f__Peptococcaceae;g__Peptococcus | 0.001852 |
| d__Bacteria;p__Proteobacteria;c__Gammaproteobacteria;o__Burkholderiales;f__Sutterellaceae;g__Sutterella | 0.001753 |
| d__Bacteria;p__Actinobacteriota;c__Actinobacteria;o__Actinomycetales;f__Actinomycetaceae;g__Mobiluncus | 0.001681 |

**Supplementary Table 5. Continue**

| **Taxa** | **Importance** |
| --- | --- |
| d__Bacteria;p__Firmicutes;c__Bacilli;o__Bacillales;f__Bacillaceae;g__Bacillus | 0.001663 |
| d__Bacteria;p__Firmicutes;c__Bacilli;o__Staphylococcales;f__Staphylococcaceae;g__Staphylococcus | 0.001653 |
| d__Bacteria;p__Firmicutes;c__Clostridia;o__Peptostreptococcales-Tissierellales;f__Peptostreptococcaceae;g__Terrisporobacter | 0.001541 |
| d__Bacteria;p__Proteobacteria;c__Gammaproteobacteria;o__Burkholderiales;f__Alcaligenaceae;g__Pelistega | 0.00142 |
| d__Bacteria;p__Proteobacteria;c__Gammaproteobacteria;o__Pseudomonadales;f__Moraxellaceae;g__Acinetobacter | 0.001407 |
| d__Bacteria;p__Firmicutes;c__Clostridia;o__Lachnospirales;f__Lachnospiraceae;g__Howardella | 0.001393 |
| d__Bacteria;p__Actinobacteriota;c__Coriobacteriia;o__Coriobacteriales;f__Atopobiaceae;g__Olsenella | 0.001342 |
| d__Bacteria;p__Bacteroidota;c__Bacteroidia;o__Bacteroidales;f__Paludibacteraceae;g__Paludibacter | 0.001288 |
| d__Bacteria;p__Firmicutes;c__Clostridia;__;__;__ | 0.00128 |
| d__Bacteria;p__Firmicutes;c__Negativicutes;o__Veillonellales-Selenomonadales;f__Selenomonadaceae;g__Anaerovibrio | 0.00125 |
| d__Bacteria;p__Firmicutes;c__Clostridia;o__Lachnospirales;f__Lachnospiraceae;g__[Eubacterium]_hallii_group | 0.001203 |
| d__Bacteria;p__Firmicutes;c__Bacilli;o__Lactobacillales;f__Aerococcaceae;g__Aerococcus | 0.001071 |
| d__Bacteria;p__Actinobacteriota;c__Coriobacteriia;o__Coriobacteriales;f__Eggerthellaceae;g__Slackia | 0.001067 |
| d__Bacteria;p__Firmicutes;c__Bacilli;o__Erysipelotrichales;f__Erysipelotrichaceae;g__Dielma | 0.000944 |
| d__Bacteria;p__Firmicutes;c__Clostridia;o__Oscillospirales;__;__ | 0.000938 |
| d__Bacteria;p__Firmicutes;c__Clostridia;o__Lachnospirales;f__Lachnospiraceae;g__Cellulosilyticum | 0.000914 |
| d__Bacteria;p__Firmicutes;c__Clostridia;o__Oscillospirales;f__Ruminococcaceae;g__Incertae_Sedis | 0.00088 |
| d__Bacteria;p__Bacteroidota;c__Bacteroidia;o__Bacteroidales;f__Prevotellaceae;g__Alloprevotella | 0.000776 |
| d__Bacteria;p__Firmicutes;c__Clostridia;o__Lachnospirales;f__Lachnospiraceae;g__Hungatella | 0.000761 |
| d__Bacteria;p__Bacteroidota;c__Bacteroidia;o__Bacteroidales;f__Prevotellaceae;g__Prevotellaceae_UCG-004 | 0.000744 |
| d__Bacteria;p__Firmicutes;c__Bacilli;o__Erysipelotrichales;f__Erysipelotrichaceae;g__Faecalicoccus | 0.000742 |
| d__Bacteria;p__Firmicutes;c__Clostridia;o__Peptostreptococcales-Tissierellales;f__Peptostreptococcaceae;g__Paeniclostridium | 0.00072 |
| d__Bacteria;p__Bacteroidota;c__Bacteroidia;o__Bacteroidales;f__Muribaculaceae;g__Muribaculaceae | 0.000709 |

**Supplementary Table 5. Continue**

| **Taxa** | **Importance** |
| --- | --- |
| d__Bacteria;p__Firmicutes;c__Clostridia;o__Peptostreptococcales-Tissierellales;f__Anaerovoracaceae;g__Family_XIII_AD3011_group | 0.000709 |
| d__Bacteria;p__Firmicutes;c__Clostridia;o__Oscillospirales;f__UCG-010;g__UCG-010 | 0.000703 |
| d__Bacteria;p__Firmicutes;c__Clostridia;o__Lachnospirales;f__Lachnospiraceae;g__Epulopiscium | 0.000668 |
| d__Bacteria;p__Firmicutes;c__Clostridia;o__Lachnospirales;f__Defluviitaleaceae;g__Defluviitaleaceae_UCG-011 | 0.000643 |
| d__Bacteria;p__Firmicutes;c__Clostridia;o__Lachnospirales;f__Lachnospiraceae;g__Acetitomaculum | 0.000631 |
| d__Bacteria;p__Bacteroidota;c__Bacteroidia;o__Bacteroidales;f__F082;g__F082 | 0.000629 |
| d__Bacteria;p__Firmicutes;c__Bacilli;o__Bacillales;f__Planococcaceae;g__Lysinibacillus | 0.000626 |
| d__Bacteria;p__Firmicutes;c__Clostridia;o__Peptostreptococcales-Tissierellales;f__Anaerovoracaceae;g__Mogibacterium | 0.000623 |
| d__Bacteria;p__Desulfobacterota;c__Desulfovibrionia;o__Desulfovibrionales;f__Desulfovibrionaceae;g__Mailhella | 0.000596 |
| d__Bacteria;p__Firmicutes;c__uncultured;o__uncultured;f__uncultured;g__uncultured | 0.000513 |
| d__Bacteria;p__Verrucomicrobiota;c__Kiritimatiellae;o__WCHB1-41;f__WCHB1-41;g__WCHB1-41 | 0.000477 |
| d__Bacteria;p__Firmicutes;c__Clostridia;o__Peptostreptococcales-Tissierellales;f__Peptostreptococcales-Tissierellales;g__Finegoldia | 0.000469 |
| d__Bacteria;p__Firmicutes;c__Clostridia;o__Oscillospirales;f__Butyricicoccaceae;g__UCG-009 | 0.000462 |
| d__Bacteria;p__Proteobacteria;c__Gammaproteobacteria;o__Burkholderiales;f__Comamonadaceae;__ | 0.000429 |
| d__Bacteria;p__Firmicutes;c__Clostridia;o__Peptostreptococcales-Tissierellales;f__Peptostreptococcaceae;g__Romboutsia | 0.000402 |
| d__Bacteria;p__Firmicutes;c__Clostridia;o__Oscillospirales;f__Ruminococcaceae;g__Angelakisella | 0.000223 |
